# Whole-Killed Blood-Stage Vaccine: Is It Worthwhile to Further Develop It to Control Malaria?

**DOI:** 10.3389/fmicb.2021.670775

**Published:** 2021-04-30

**Authors:** Jingjing Cai, Suilin Chen, Feng Zhu, Xiao Lu, Taiping Liu, Wenyue Xu

**Affiliations:** ^1^College of Basic Medicine, Army Medical University (Third Military Medical University), Chongqing, China; ^2^Department of Pathogenic Biology, Army Medical University (Third Military Medical University), Chongqing, China; ^3^Key Laboratory of Extreme Environmental Medicine, Ministry of Education of China, Chongqing, China; ^4^Department of Thoracic Surgery, Xinqiao Hospital, Army Medical University (Third Military Medical University), Chongqing, China

**Keywords:** malaria parasite, whole-killed blood-stage vaccine, liver stage, sexual-stage, blood stage

## Abstract

Major challenges have been encountered regarding the development of highly efficient subunit malaria vaccines, and so whole-parasite vaccines have regained attention in recent years. The whole-killed blood-stage vaccine (WKV) is advantageous as it can be easily manufactured and efficiently induced protective immunity against a blood-stage challenge, as well as inducing cross-stage protection against both the liver and sexual-stages. However, it necessitates a high dose of parasitized red blood cell (pRBC) lysate for immunization, and this raises concerns regarding its safety and low immunogenicity. Knowledge of the major components of WKV that can induce or evade the host immune response, and the development of appropriate human-compatible adjuvants will greatly help to optimize the WKV. Therefore, we argue that the further development of the WKV is worthwhile to control and potentially eradicate malaria worldwide.

## Introduction

Malaria remains a potentially fatal public health problem, resulting in high morbidity and mortality in tropical and subtropical regions. Recently, malaria control interventions, such as artemisinin-based combination therapy (ACT), insecticide-treated bed nets (ITNs), and other mosquito vector control strategies, have greatly reduced the incidence of malaria all over the world (O’Meara et al., 2010). However, the emergence of artemisinin derivative-resistant malaria parasites (Phyo et al., 2012; Uwimana et al., 2020) and insecticide-resistant mosquitoes (Ranson et al., 2011) has greatly hampered the effectiveness of ACTs and ITNs. Therefore, the development of a highly efficient malaria vaccine has been regarded as the most cost-effective tool for malaria control and elimination, and potentially even eradication (Crompton et al., 2010).

**Figure 1:**
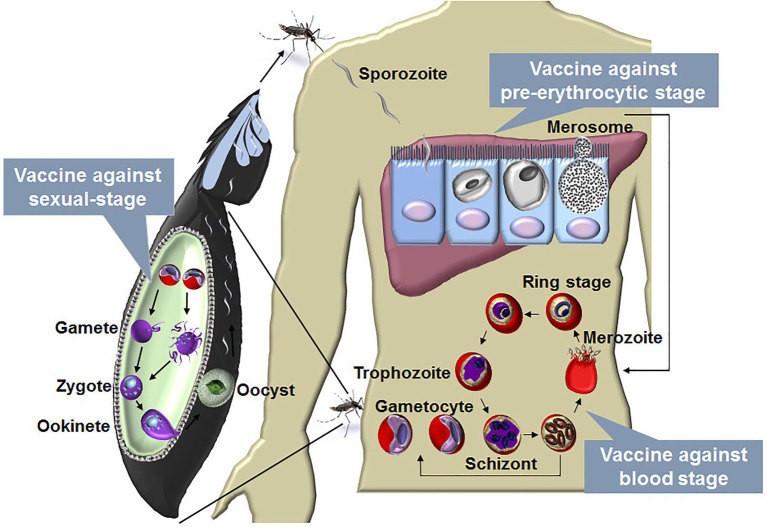
The life cycle of malaria parasite and vaccines designed against each stage. The life cycle of *Plasmodium* includes liver stage and blood stage in human and sexual-stage in the mosquito vector. A *Plasmodium* infection begins when an infected female *Anopheles* mosquito takes a blood meal and injects a small number of sporozoites into a human host. The sporozoites enter blood vessels and invade target hepatocytes, with a single sporozoite giving rise to tens of thousands of merozoites. Next, merozoites are budded from an infected hepatocyte by the merosomes and rupture to release thousands of merozoites into the bloodstream. In the bloodstream, the merozoites invade RBCs, initiating blood-stage development. A merozoite, which subsequently develop into ring, trophozoite, and schizont stage parasites, can multiply up to 20-fold every 1 to 3 days in cycles of invasion, replication, and RBC rupture. New daughter merozoites are released and rapidly invade the non-infected erythrocytes. Within erythrocytes, some of the merozoites differentiate into sexual forms of the parasite, which are called as male or female gametocytes. When male and female gametocytes are picked up by a female *Anopheles* mosquito during a blood meal, they develop further into mature sexual stages called as gametes. Fertilization happens between the male and female gametes, giving rise to a zygote. The developing zygotes transform into elongated motile ookinetes, which invade through the midgut wall of the mosquito and form oocysts on the exterior surface. Once the oocysts have matured, they eventually burst, releasing thousands of sporozoites that migrate to the mosquito salivary glands ready to infect another human host during the next mosquito blood meal (Julien and Wardemann, 2019). A vaccine against the blood stage aims to reduce the mortality and morbidity of malaria patients, and vaccine against the liver stage is a promising method to prevent malaria infection. The development of the parasite in the vector is essential for transmission, a vaccine against the sexual-stage aims, therefore, to block transmission of malaria parasites.

Malaria is caused by the infections of species in the *Plasmodium* genus. There are four human malaria parasites, namely, *Plasmodium vivax* (*P. vivax*), *P. falciparum*, *P. ovale*, and *P. malariae*. Of these, *P. falciparum* is the deadliest. A *Plasmodium* infection is initiated by sporozoite inoculation into the host skin by an infected female *Anopheles* mosquito. The sporozoites travel to the liver and infect a small number of hepatocytes, with a single sporozoite giving rise to tens of thousands of merozoites. Next, merozoites are released into the bloodstream when merosomes bud from an infected hepatocyte, and they invade red blood cells (RBCs), initiating blood-stage development. A merozoite can multiply up to 20-fold every 1–3 days in cycles of invasion, replication, and RBC rupture, which releases many infectious merozoites. Some of the asexual blood stages transform into sexual forms called as gametocytes, which can eventually move into the midgut of a blood-feeding mosquito and then develop into sporozoites in the salivary gland. The blood-feeding mosquito then injects these sporozoites into another human, thereby initiating a new human infection (Figure 1).

Thus, the *Plasmodium* life cycle involves the liver stage (pre-erythrocytic stage) and blood stage in mammals and the sexual-stage in its vector. The blood stage is the main cause of the clinical manifestations (ranging from mild to severe malaria), and a blood-stage vaccine aims to reduce mortality and morbidity in malaria patients. In contrast, the liver stage is clinically silent, and a liver-stage vaccine is promising for preventing malaria infection (Smith et al., 2012). The development of the parasite in mosquitoes is essential for malaria transmission. Therefore, a sexual-stage vaccine aims to block malaria transmission (Acquah et al., 2019; Figure 1). For each stage, two kinds of vaccines, namely subunit and whole-parasite vaccines, are being explored. As major challenges have been encountered regarding subunit malaria vaccines, whole-parasite vaccines have recently been regaining attention. Here, we discuss promising aspects regarding the development of a whole-killed blood-stage vaccine (WKV) that protects against all stages and perspectives on its optimization in the future.

## Major Challenges Have Been Encountered in the Development of a Highly Efficient Subunit Malaria Vaccine

In the past three decades, great efforts have been made to develop efficient subunit vaccines against the pre-erythrocytic, blood, and sexual-stages, but major challenges have been encountered. The dominant protective antigen of the pre-erythrocytic stage is the main surface protein of sporozoites, circumsporozoite protein (CSP), which plays an important role in sporozoite invasion of hepatocytes (Dame et al., 1984; Rathore et al., 2002; Kumar et al., 2006). The most advanced CSP-based subunit malaria vaccine is RTS,S/AS01. This vaccine consists of a large part of the CSP fused with hepatitis B virus surface antigen (HBV-S Ag) particle formulated with the potent liposomal adjuvant AS01 (Cohen, 1996; Casares et al., 2010). Phase 3 clinical trials showed that the vaccine’s protection efficacy was 50.4% against the first clinical episode of malaria and 45.1% against severe malaria in young children, but only 30.1% in infants (Agnandji et al., 2011, 2012). In 2015, the European Medicines Agency announced the adoption of a positive scientific opinion regarding the use of RTS,S in regions outside of the European Union, where malaria is a major problem (EMA, 2015). In 2019, the World Health Organization (WHO) recommended the large-scale pilot implementations of routine use of RTS,S in real-life settings, involving moderate-to-high malaria transmission in order to assess its protective benefits and safety (WHO, 2019). However, RTS,S/AS01 still does not meet the criteria for a licensable first-generation vaccine (50% efficacy lasting for ≥1 year; Crompton et al., 2010), as the vaccine efficacy was dropped to 28 and 18% at 3–4 years post-vaccination in children and young infants, respectively (Rts, 2015). Therefore, the main challenge of the use of RTS,S is to sustain high antibody concentrations to mediate durable protection.

Most blood-stage candidate antigens are essential invasion proteins of merozoites such as merozoite surface protein (MSP1) and MSP3, apical membrane antigen 1 (AMA1), and erythrocyte-binding antigen 175 (EBA-175). These blood stage malaria vaccine candidates seek to induce high titers of plasmodium specific antibody that inhibit erythrocyte invasion by merozoites or limit parasite replication in red blood cells. However, most of the vaccine candidates exhibit extensive polymorphism between malaria strains from different geographical regions, and immunization with these vaccines only led to partial protection against vaccine-like strains (Bull et al., 1998; Sutherland, 2007; Fowkes et al., 2010). In addition, the RBC invasion pathways of merozoite exhibit redundancy, with the blockade of one invasion pathway failing to confer protection against challenge (Ogutu et al., 2009; Sagara et al., 2009; Sirima et al., 2011). The leading vaccine candidates AMA1 did not provide significant protection against clinical malaria in vaccine trials, and an MSP3 vaccine in Burkinabe children also demonstrated short-term protection (Sirima et al., 2011; Thera et al., 2011). At present, the most promising blood-stage candidate antigen is *P. falciparum* reticulocyte-binding protein homolog 5 (PfRH5), which interacts with basigin (CD147) on the RBC surface during merozoite invasion (Wright et al., 2014). PfRH5 exhibits limited polymorphism, and PfRH5/basigin is essential for merozoite invasion of RBCs (Weiss et al., 2015; Volz et al., 2016). Pre-clinical studies showed that PfRH5 induced cross-strain neutralizing antibody (Douglas et al., 2011) and confers nearly complete protection against challenge with heterologous *Plasmodium* strains in Aotus monkeys (Douglas et al., 2015). Recent study has defined that the neutralizing and non-neutralizing epitopes of PfRH5 are both essential for red blood cell invasion by the merozoites, which will optimize the designation of PfRH5-based vaccine (Alanine et al., 2019). However, protection in monkeys required a high level of anti-PfRH5 IgG (an estimated 200 μg/ml), and there was modest or no boosting of vaccine-induced antibody by infection of the monkeys (Douglas et al., 2015). This may limit the efficacy and duration of protection conferred by a PfRH5-based subunit vaccine.

Leading candidate antigens related to the malaria sexual-stage, include *P. falciparum* Pfs230 and Pfs48/45, which are expressed by gametocytes, and Pfs25, which is exclusively expressed by zygotes and ookinetes. In phase 1 clinical trials, vaccines based on Pfs25 and its *P. vivax* ortholog Pvs25 have induced antibodies that block mosquito infection (Wu et al., 2008). However, substantial antibody levels were only achieved after four doses, and antibody levels rapidly waned after the final dose (Sagara et al., 2018). In addition, these antigens are cysteine-rich with multiple 6-cys domains and/or epidermal growth factor (EGF)-like domains, and it is difficult to prepare the properly folded recombinant protein (Barr et al., 1991).

The major issue regarding the current subunit malaria vaccines is that they always contain only a single or a small number of antigens, which do not induce broad-spectrum long-lasting immune responses. It is well known that *Plasmodium* encodes approximately 5,300 proteins, but only a few candidate antigens have been identified (Gardner et al., 2002; Doolan et al., 2003). Therefore, it is currently difficult to design highly efficient subunit malaria vaccines.

## Promising Malaria Vaccine: Whole-Parasite Blood-Stage Vaccine

In contrast to the narrow antigen spectrum related to subunit vaccines, whole-parasite vaccines maximize the spectrum of antigens, greatly enhancing protective immunity, and they have attracted more attention in recent years (Tarun et al., 2008; Ting et al., 2008; Moorthy and Ballou, 2009; Aly et al., 2010; Demarta-Gatsi et al., 2016; Mordmuller et al., 2017; Table 1). Attenuated sporozoites are the most promising vaccines against the pre-erythrocytic stage. It has been reported that either irradiation-, genetic-, or chemo-attenuated sporozoites (also called as chemoprophylaxis with sporozoites, CPS) could induce sterilizing protective immunity against homologous sporozoite challenge in both mice and humans (Nussenzweig et al., 1967; Mueller et al., 2005; Roestenberg et al., 2009; Epstein et al., 2011; Bijker et al., 2013; Seder et al., 2013). An irradiation-attenuated sporozoite vaccine could also induce a strain-transcending T cell response and durable protection against heterologous controlled human malaria infection (CHMI; Lyke et al., 2017). PfSPZ vaccine was confirmed to be extremely well tolerated and showed the significant protection in Mali adults against *P falciparum* infection in phase 1 trial (Sissoko et al., 2017). Furthermore, in the recent study by Roestenberg et al. (2020) in phase 1/2a trial, genetically attenuated malaria vaccine PfSPZ-GA1 also showed the modest protective immunity to mosquito-bite challenge. However, safety remains a great concern because the breakthrough of genetically attenuated sporozoites has been reported (Vaughan et al., 2010; Spring et al., 2013). Meanwhile, sporozoites can only be developed in mosquitoes, and sourcing and delivering aseptic, purified, and cryopreserved sporozoites limit the broad application of sporozoite vaccines (Prinz et al., 2018).

**Table 1:** The time table of the development of whole parasite vaccines.

Parasite stage and vaccine candidate	Time	Current status
**Pre-erythrocytic stage**		
Radiation-attenuated sporozoite vaccine (rodent)	1967 (Nussenzweig et al., 1967)	Preclinical
Chemically attenuated sporozoite vaccine	2009 (Roestenberg et al., 2009)	Phase 2
Genetically attenuated sporozoite vaccine	2005 (Mueller et al., 2005)	Phase 1
Radiation-attenuated sporozoite vaccine (human)	2011 (Clyde et al., 1973; Clyde, 1975; Epstein et al., 2011b)	Phase 2
**Blood stage**		
Whole-killed blood-stage vaccine	1948 (Freund et al., 1948)	Preclinical
Genetic-attenuated whole-parasite blood-stage vaccines	2008 (Ting et al., 2008)	Preclinical
Chemically attenuated whole-parasite blood-stage vaccines	2018 (Stanisic et al., 2018)	Phase 1

Pre-erythrocytic and blood-stage whole parasite vaccines are being evaluated in clinical trials (denoted as phases 1–4) or are being tested in rodent or non-human primate models (preclinical research).

Compared to the sourcing of sporozoites, the culture of the *Plasmodium falciparum* blood stage is a well-established process, so whole-parasite blood-stage vaccines can easily be manufactured. Therefore, efforts have been made to develop genetically or chemically attenuated whole-parasite blood-stage vaccines. Attenuated *P. yoelii* (*Plasmodium yoelii*) with purine nucleoside phosphorylase (PNP), nucleoside transporter 1 (NT1), or histamine-releasing factor (HRF) deficiency confer complete sterile protection against both homologous and heterologous blood-stage challenge (Ting et al., 2008; Aly et al., 2010; Demarta-Gatsi et al., 2016). Regarding chemical attenuation, the blood stage can be chemically attenuated both *in vivo* and *in vitro*. *In vivo*, this has been achieved by creating a persistent subpatent blood-stage infection using a low dose of malaria parasite followed by treatment with anti-malaria drugs, which induces sterile protection against both high-dose homologous and heterologous blood-stage challenge (Pombo et al., 2002; Elliott et al., 2005). *In vitro*, several approaches have been used to chemically attenuate the blood stage, such as treatment with the parasite DNA-binding drugs centanamycin (CM; Good et al., 2013) and tafuramycin-A (TF-A; Stanisic et al., 2018), or the delayed death-causing drugs doxycycline or azithromycin (Low et al., 2019). The vaccination of *in vitro* chemically attenuated blood stage has also been reported to induce protective immunity against both homologous and heterologous blood-stage challenges (Good et al., 2013; Raja et al., 2016; Low et al., 2019). However, safety concerns regarding the breakthrough of genetically or chemically attenuated blood stages remains unavoidable. In contrast, a WKV, which is an inactive vaccine prepared by several cycles of freezinge/thawing of pRBCs (mostly containing schizonts), is theoretically much safer than other forms of whole-parasite blood-stage vaccine. Furthermore, our previous study and other research have demonstrated that the WKV induced strong protective immunity against both homologous and heterologous blood-stage challenge (Liu et al., 2013; Lu et al., 2017).

Most importantly, the protection brought about by genetically or chemically attenuated blood-stage vaccines involved both antibodies and CD4^+^ T cell responses (Good et al., 2013; Raja et al., 2016; Low et al., 2019). However, WKV with CpG as the adjuvant is dependent on CD4^+^ T cell responses, which might target the antigens that are conserved across strains (Pinzon-Charry et al., 2010). Thus, whole-parasite blood-stage vaccines, such as WKV, induce strong CD4^+^ T cell responses against universal epitopes, allowing them to confer species-transcending protection, unlike most subunit malaria vaccines against the polymorphic B cell epitopes.

**Figure 2:**
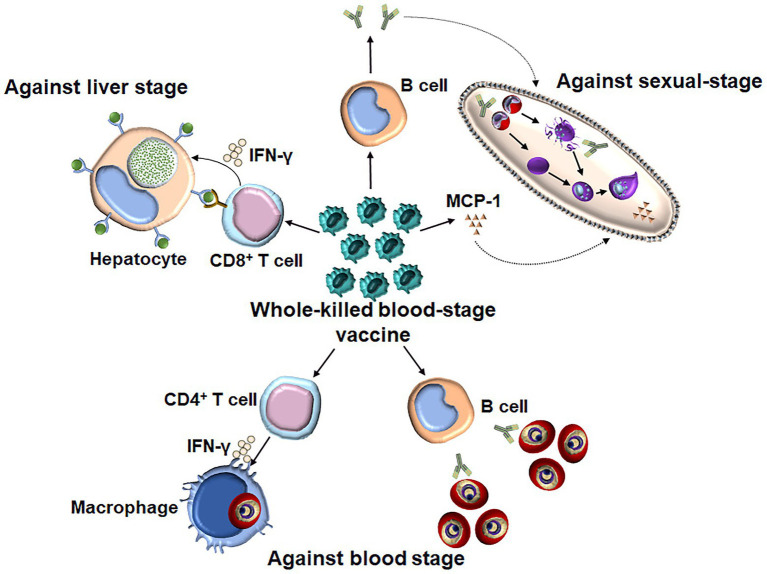
The underlying mechanism of WKV against all three stages. WKV (an inactivate vaccine) could not only efficiently induce a protection against a blood-stage challenge but also confer the cross-stage protection against both liver stage and sexual-stage. The protective immunity against blood stage is dependent on both parasite-specific antibody and CD4^+^ T cells, which might enhance the capacity of macrophages to kill the intracellular parasite through the secretion of IFN-γ. WKV could also provide cross-protection against liver stage through priming parasite-specific CD8^+^ T cells, although the target antigen of which is still needed to be defined. In contrast, the protective immunity induced by WKV against sexual-stage is largely dependent on parasite-specific antibody and MCP-1, the non-specific effect of latter might explain the species-transcending protection against sexual-stage.

It is thought that a subunit vaccine developed to induce a response against a certain stage of the malaria parasite induces stage-specific, but not cross-stage, immunity. In contrast, whole-parasite vaccines against the pre-erythrocytic stage, such as chemically and genetically attenuated sporozoites, have been shown to induce cross-stage protection against the blood stage (Nahrendorf et al., 2015b; Sack et al., 2015). Although the protection induced by chemically attenuated blood-stage *P. yoelii* parasites was stage specific as immunized mice were not protected against intravenous or mosquito bite sporozoite challenges (Raja et al., 2016), our study showed that the WKV not only conferred the cross-stage immunity against sporozoite challenge but also blocked parasite development in mosquitoes (Figure 2; Lu et al., 2017; Zhu et al., 2017). Therefore, the WKV not only prevents malarial infection and reduces mortality and morbidity among malaria patients but also blocks malaria transmission. Compared to the genetically or chemically attenuated blood-stage vaccines, the WKV is more acceptable and easily manufactured and shows promise regarding controlling and even eradicating malaria worldwide.

## Optimization of the WKV

The main limitation of the WKV is the high dose required per vaccine due to its low immunogenicity. The immunization dose of WKV typically needs to be at least 1.5 × 10^7^ parasites or parasite equivalents per vaccine dose (Liu et al., 2013). Therefore, it is a significant challenge due to the difficulties in vaccine manufacture, which involves the human red blood cells. The use of human blood products may also have the possibility to contaminate the vaccine with infectious adventitious agents in the manufacturing process. In the final WKV formulation, an appropriate human-compatible adjuvant is required to induce protection with the lowest dose of parasite. Up to now, all the whole-parasite blood-stage vaccines are at a very early stage of clinical evaluation, and there is no WKV enters clinical trial for the assessment of safety and immunogenicity (Stanisic et al., 2018). Hence, the clinical evaluation of WKV remains a significant challenge. Additionally, the safety concerns regarding the WKV should also be considered, as the parasite is grown in human RBCs, and immunization with pRBCs may induce anti-RBC antibodies and possibly lead to autoimmunity following immunization (Good, 2011). Although no adverse events have been reported, the immunogenicity of the current WKV still needs to be improved. One way to potentially do this is to gain further understanding into the underlying mechanisms of the WKV against each of the three stages, and another is to develop human-compatible adjuvants.

### Understanding the Mechanisms of the WKV Against All Three Stages

Knowledge of the mechanisms of the WKV against all stages would guide the optimization of its formulation. Regarding the protective immunity induced by the WKV against the blood stage, several studies have demonstrated that it is dependent on both humoral and CD4^+^ T cell responses (Pinzon-Charry et al., 2010). Although the purine salvage enzyme HGXPT (hypoxanthine guanine xanthine phosphoribosyl transferase) has been identified as a major potential target antigen for CD4^+^ T cell responses against the blood stage (Makobongo et al., 2003; Woodberry et al., 2009), the protective B cell antigens of WKV still need to be defined. We found that the WKV could greatly increase the frequency of CD8α^low^CD11a^high^ T cells, which are representative malaria parasite-specific CD8^+^ T cells (Rai et al., 2009). A T cell depletion assay demonstrated that the protective immunity induced by the WKV against the liver stage is mainly dependent on CD8^+^ T cell responses, but not CD4^+^ T cell responses (Lu et al., 2017). Cytotoxic CD8^+^ T cells were previously thought to have no role against the blood stages because RBCs generally do not express human leukocyte antigen class I (HLA I). However, a recent study showed that *P. vivax*-infected reticulocytes express HLA I and circulating CD8^+^ T cells in malaria patients can recognize the *P. vivax*-infected reticulocytes in a HLA-dependent manner and kill the intracellular parasites (Junqueira et al., 2018). Despite this, the relative importance of the role of parasite-specific CD8^+^ T cells regarding the protective immunity induced by the WKV against the *Plasmodium*, which cannot infect reticulocytes, remains unclear. In contrast to protective immunity against the blood and liver stages, we found that CD4^+^ T effector cells are dispensable for the cross-protection induced by the WKV against the sexual-stage (Zhu et al., 2017). Instead, this cross-protection is mainly dependent on malaria parasite-specific antibodies and monocyte chemoattractant protein (MCP)-1, but not interferon (IFN)-γ (Zhu et al., 2017). Although the exact mechanism of MCP-1 against the sexual-stage in this context is unknown, its non-specific effect might interpret the cross-species protection induced by the WKV against sexual-stage.

Interestingly, the cellular and humoral immune responses induced by the WKV are critical for its cross-protection against the liver and sexual-stage, respectively (Liu et al., 2013; Lu et al., 2017). Therefore, the investigation of underlying mechanisms for the activation of cellular and humoral immune responses may help us to optimize the WKV against all stages. It has been shown that the activation of malaria parasite-specific immune responses during WKV immunization involves malarial hemozoin triggering TLR9 (Toll-like receptor 9; Coban et al., 2010). Our previous data also indicated that the C5a/C5aR signaling pathway in dendritic cells (DCs) was activated during immunization and is essential for the optimal induction of the malaria parasite-specific CD4^+^ T cell response (Liu et al., 2013). However, the investigation of the critical components of the WKV responsible for the upregulation of protective immune responses has only just begun.

**Figure 3:**
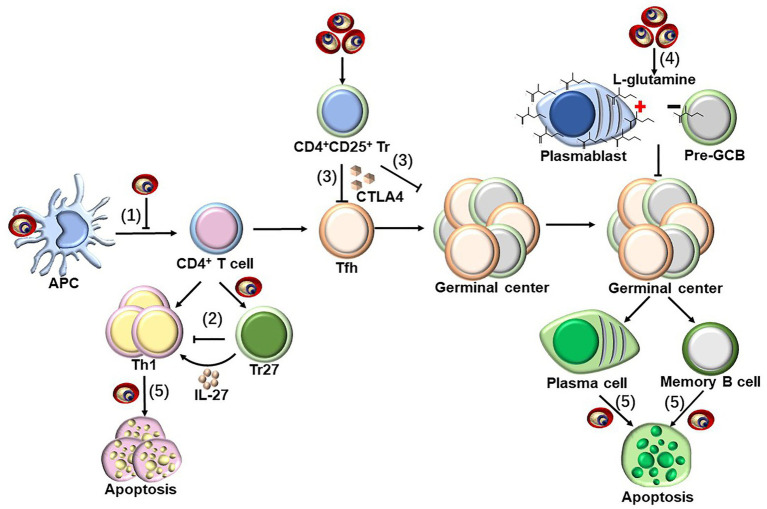
Strategies to suppress host immune response by blood-stage malaria parasites. Parasite-specific CD4^+^ T cells are essential for host protective immunity against blood stage, as which can not only directly clear parasite through secretion Th1 cytokines but also provide help for B cells to produce antibody. However, malaria parasites have evolved to evade host immune response through following strategies. (1) pRBCs could inhibit the maturation of DCs, and subsequently suppress the CD4^+^ T cell activation (Urban et al., 1999; Woodberry et al., 2012; Pinzon-Charry et al., 2013). (2) Tr27 cells, the IL-27-producing CD4^+^ T cells, have also been reported to be activated during malaria infection, which reciprocally suppresses CD4^+^ T cells activation through the secretion of IL-27 (Kimura et al., 2016). (3) Malaria infection also induce the expansion of CD4^+^CD25^+^ regulatory T cells, which interfere T helper cell responses and follicular T helper (TFH)-B cell interactions in germinal centers through secretion of CTLA-4 (Kurup et al., 2017). (4) The early differentiation of parasite-specific B cells into short-lived plasmablasts consumes a large amount of L-glutamine during malaria blood-stage infection, resulting in the nutrient deprivation of GCB, limiting the generation of MBC and LLPC responses against malaria parasites (Vijay et al., 2020). (5) The infection of malaria parasite could even induce the apoptosis of the activated CD4^+^ T cells dependent on IFN-γ, but both LLPCs and MBCs are deleted by an unknown mechanism (Hirunpetcharat and Good, 1998; Wipasa et al., 2001; Xu et al., 2002; Wykes et al., 2005).

In addition to the positive regulatory components, the identification and genetic deletion of the components that negatively regulate the protective immune response is also critical for vaccine optimization. In humans, the malaria parasite induces DC apoptosis and thereby inhibits parasite-specific CD4^+^ T cell responses to facilitate its survival (Woodberry et al., 2012; Pinzon-Charry et al., 2013), along with downregulating costimulatory molecules on DCs (Urban et al., 1999). Additionally, Tr27 cells, which are IL-27-producing CD4^+^ T cells, are induced by the malaria parasite to regulate CD4^+^ T cell responses against the infection (Kimura et al., 2016). The expansion of CD4^+^CD25^+^ regulatory T cells during malaria infection suppresses T helper cell responses and follicular T helper (TFH)-B cell interactions in germinal centers *via* secretion of CTLA-4 (Figure 3; Kurup et al., 2017). This has been confirmed by the findings that the protection efficacy induced by several whole-parasite vaccines is much lower in malaria-exposed African individuals than in malaria-naïve United States and European individuals, and the malaria blood stage infection also suppresses both the humoral and cellular immune responses against sporozoites (Ocana-Morgner et al., 2003; Keitany et al., 2016). Furthermore, durable immunity was also difficult to be induced in African individuals, potentially because malaria inhibits the vaccine-induced formation of long-lived plasma cells (LLPCs) and high-affinity memory B cells (MBCs). Multiple mechanisms have been postulated to explain the insufficient induction of LLPCs and MBCs, including preferential expansion of CXCR3^+^ (TH1-like) TFH cells (Obeng-Adjei et al., 2015), regulatory T cells (Kurup et al., 2017), and atypical MBCs (Weiss et al., 2009; Obeng-Adjei et al., 2017), as well as the dysregulation of chemokines and cytokines (Ryg-Cornejo et al., 2016) and the induction of immune checkpoints (Butler et al., 2011), which may delay or impair the acquisition of humoral immunity against malaria. In addition, a recent study revealed that the metabolic hyperactivity of plasmablasts resulted in nutrient deprivation that affects the germinal center reaction, limiting the generation of MBC and LLPC responses against malaria parasites (Vijay et al., 2020). The limited duration of the host protective immunity against the malaria parasite may also be explained by the parasite, inducing the apoptosis of LLPCs, MBCs, and activated CD4^+^ T cells (Figure 3; Hirunpetcharat and Good, 1998; Wipasa et al., 2001; Xu et al., 2002; Wykes et al., 2005). Lastly, the immune evasion mechanisms established by the malaria blood stage also include the parasite antigen variation, sequestration, and rosetting (Niang et al., 2014; Wahlgren et al., 2017; Belachew, 2018).

The identification of cross-protective antigens shared by all three stages could also help to optimize the multi-stage effects of the WKV and facilitate the development of a multi-stage malaria subunit vaccine. Several recent studies have reported cross-stage protection between the blood and liver stages (Nahrendorf et al., 2015b; Sack et al., 2015), and the identification of cross-protective antigens has attracted more attention (Nahrendorf et al., 2015a). Although no cross-protective antigens have yet been identified, it should be feasible to screen for the overlapping antigens by comparing the transcriptional profiles between two stages. Additionally, the proteome-wide screening of antibody and T cell reactivity between protected and unprotected individuals (immunomics) may also help to characterize the cross-protective antigens (Doolan, 2011; Davies et al., 2015; Schussek et al., 2017). Concurrently, further research is needed on WKV components that may downregulate host protective immune responses.

### Development of an Appropriate Human-Compatible Adjuvant

Developing an appropriate human-compatible adjuvant to enhance the immunogenicity of the WKV is an urgent issue. Aluminum-containing adjuvants are the most widely used clinical adjuvants, which have been demonstrated not only to directly trigger the NALP3 inflammasome but also to indirectly activate the host innate immune response by inducing the release of the endogenous danger signal uric acid (Lambrecht et al., 2009). However, we found that alum only slightly improved the immunogenicity of pRBC lysate (Fu et al., 2020). Although CpG has been reported to be able to significantly enhance the malaria parasite-specific CD4^+^ T responses and reduce the vaccine dose from 10^8^ to 10^3^ (Pinzon-Charry et al., 2010), the use of CpG as an adjuvant in humans has not been approved.

The immunomodulatory role of chloroquine has been known for approximately 30 years. Early *ex vivo* studies claimed that chloroquine had a direct suppressive effect on several immune cells, such as T cells and natural killer cells (Bygbjerg et al., 1986, 1987; Goldman et al., 2000). However, chloroquine was found to greatly promote CD8^+^ T cell responses against soluble antigens *in vivo*, through inhibiting the degradation of internalized soluble antigen in endosomes by increasing the pH of the endosomes (Accapezzato et al., 2005). Interestingly, we found that low-dose antimalarial chloroquine along with alum synergistically improved the immunogenicity of pRBC lysates by enhancing the humoral response, although chloroquine alone only had a slight effect (Fu et al., 2020). As chloroquine has been approved as safe for humans, we strongly suggest that the use of low-dose chloroquine, along with alum, should be explored to enhance the immunogenicity of WKV.

## Concluding Remarks and Future Perspectives

Although the burden of malaria has been greatly reduced by control measures in the past decades, a highly efficient and safe vaccine is a high priority to ultimately control and even eradicate malaria around the world. *Plasmodium* is a complicated organism, encoding more than 5,000 proteins, but only a few protective antigens have been identified. Therefore, the current subunit vaccine strategy has encountered major challenges, as these vaccines can only induce narrow immune responses, and the future development of malaria subunit vaccine is not optimistic. Although multi-stage or multi-antigen vaccines are theoretically promising to control malaria, it is dependent on the identification of protective antigens. In contrast, the immune responses induced by whole-parasite vaccines are broad, sparking a renewed interest in these vaccines. Owing to the difficulties in sourcing and delivering cryopreserved sporozoites, whole-parasite blood-stage vaccine manufacturing is more feasible, as the culture of *P. falciparum* blood stage is a well-established process. Furthermore, the *Plasmodium* life cycle is very complicated, with three developmental stages, each of which has different antigens. An ideal vaccine would confer cross-stage immunity against all three stages, and WKV, an inactive whole-parasite vaccine, is safe and can induce cross-protection against both the liver and sexual-stages.

However, the immunogenicity of WKV is low and needs to be improved. To optimize WKV, two major elements should be identified: (1) malaria parasite components that positively regulate the host immune responses and (2) the mechanisms and parasite components that suppress or evade the host immune responses, which would affect the magnitude and duration of the protective immune response induced by WKV. With this knowledge, negative regulatory components could be removed and positively components could be strengthened from the parasite by genetic manipulation to greatly improve the immunogenicity and protective efficacy of the WKV.

## Author Contributions

WX and TL: conceptualization, writing-review and editing, and funding acquisition. JC, SC, XL, and FZ: writing-original draft. All authors contributed to the article and approved the submitted version.

### Conflict of Interest

The authors declare that the research was conducted in the absence of any commercial or financial relationships that could be construed as a potential conflict of interest.

## References

[ref1] Accapezzato D. Visco V. Francavilla V. Molette C. Donato T. Paroli M.. (2005). Chloroquine enhances human CD8+ T cell responses against soluble antigens in vivo. J. Exp. Med. 202, 817–828. 10.1084/jem.20051106, PMID: 16157687 PMC2212941

[ref2] Acquah F. K. Adjah J. Williamson K. C. Amoah L. E. (2019). Transmission-blocking vaccines: old friends and new prospects. Infect. Immun. 87, e00775–e00818. 10.1128/IAI.00775-18, PMID: 30962400 PMC6529669

[ref3] Agnandji S. T. Lell B. Fernandes J. F. Abossolo B. P. Methogo B. G. Kabwende A. L.. (2012). A phase 3 trial of RTS,S/AS01 malaria vaccine in African infants. N. Engl. J. Med. 367, 2284–2295. 10.1056/NEJMoa1208394, PMID: 23136909 PMC10915853

[ref4] Agnandji S. T. Lell B. Soulanoudjingar S. S. Fernandes J. F. Abossolo B. P. Conzelmann C.. (2011). First results of phase 3 trial of RTS,S/AS01 malaria vaccine in African children. N. Engl. J. Med. 365, 1863–1875. 10.1056/NEJMoa1102287, PMID: 22007715

[ref5] Alanine D. G. W. Quinkert D. Kumarasingha R. Mehmood S. Donnellan F. R. Minkah N. K.. (2019). Human antibodies that slow erythrocyte invasion potentiate malaria-neutralizing antibodies. Cell 178, 216.e221–228.e221. 10.1016/j.cell.2019.05.025, PMID: 31204103 PMC6602525

[ref6] Aly A. S. Downie M. J. Mamoun C. B. Kappe S. H. (2010). Subpatent infection with nucleoside transporter 1-deficient Plasmodium blood stage parasites confers sterile protection against lethal malaria in mice. Cell. Microbiol. 12, 930–938. 10.1111/j.1462-5822.2010.01441.x, PMID: 20088947

[ref7] Barr P. J. Green K. M. Gibson H. L. Bathurst I. C. Quakyi I. A. Kaslow D. C. (1991). Recombinant Pfs25 protein of *Plasmodium falciparum* elicits malaria transmission-blocking immunity in experimental animals. J. Exp. Med. 174, 1203–1208. 10.1084/jem.174.5.1203, PMID: 1940798 PMC2118997

[ref8] Belachew E. B. (2018). Immune response and evasion mechanisms of *Plasmodium falciparum* parasites. J. Immunol. Res. 2018:6529681. 10.1155/2018/6529681, PMID: 29765991 PMC5889876

[ref9] Bijker E. M. Bastiaens G. J. Teirlinck A. C. van Gemert G. J. Graumans W. van de Vegte-Bolmer M.. (2013). Protection against malaria after immunization by chloroquine prophylaxis and sporozoites is mediated by preerythrocytic immunity. Proc. Natl. Acad. Sci. U. S. A. 110, 7862–7867. 10.1073/pnas.1220360110, PMID: 23599283 PMC3651438

[ref10] Bull P. C. Lowe B. S. Kortok M. Molyneux C. S. Newbold C. I. Marsh K. (1998). Parasite antigens on the infected red cell surface are targets for naturally acquired immunity to malaria. Nat. Med. 4, 358–360. 10.1038/nm0398-358, PMID: 9500614 PMC3836255

[ref11] Butler N. S. Moebius J. Pewe L. L. Traore B. Doumbo O. K. Tygrett L. T.. (2011). Therapeutic blockade of PD-L1 and LAG-3 rapidly clears established blood-stage Plasmodium infection. Nat. Immunol. 13, 188–195. 10.1038/ni.2180, PMID: 22157630 PMC3262959

[ref12] Bygbjerg I. C. Svenson M. Theander T. G. Bendtzen K. (1987). Effect of antimalarial drugs on stimulation and interleukin 2 production of human lymphocytes. Int. J. Immunopharmacol. 9, 513–519. 10.1016/0192-0561(87)90027-0, PMID: 3497888

[ref13] Bygbjerg I. C. Theander T. G. Andersen B. J. Flachs H. Jepsen S. Larsen P. B. (1986). In vitro effect of chloroquine, mefloquine and quinine on human lymphocyte proliferative responses to malaria antigens and other antigens/mitogens. Trop. Med. Parasitol. 37, 245–247. PMID: 3538350

[ref14] Casares S. Brumeanu T. D. Richie T. L. (2010). The RTS,S malaria vaccine. Vaccine 28, 4880–4894. 10.1016/j.vaccine.2010.05.033, PMID: 20553771

[ref15] Clyde D. F. (1975). Immunization of man against falciparum and vivax malaria by use of attenuated sporozoites. Am. J. Trop. Med. Hyg. 24, 397–401. 10.4269/ajtmh.1975.24.397, PMID: 808142

[ref16] Clyde D. F. McCarthy V. C. Miller R. M. Hornick R. B. (1973). Specificity of protection of man immunized against sporozoite-induced falciparum malaria. Am. J. Med. Sci. 266, 398–403. 10.1097/00000441-197312000-00001, PMID: 4590095

[ref17] Coban C. Igari Y. Yagi M. Reimer T. Koyama S. Aoshi T.. (2010). Immunogenicity of whole-parasite vaccines against *Plasmodium falciparum* involves malarial hemozoin and host TLR9. Cell Host Microbe 7, 50–61. 10.1016/j.chom.2009.12.003, PMID: 20114028

[ref18] Cohen J. (1996). *Vaccine Composition Against Malaria*. U.S. patent.

[ref19] Crompton P. D. Pierce S. K. Miller L. H. (2010). Advances and challenges in malaria vaccine development. J. Clin. Invest. 120, 4168–4178. 10.1172/JCI44423, PMID: 21123952 PMC2994342

[ref20] Dame J. B. Williams J. L. McCutchan T. F. Weber J. L. Wirtz R. A. Hockmeyer W. T.. (1984). Structure of the gene encoding the immunodominant surface antigen on the sporozoite of the human malaria parasite *Plasmodium falciparum*. Science 225, 593–599. 10.1126/science.6204383, PMID: 6204383

[ref21] Davies D. H. Duffy P. Bodmer J. L. Felgner P. L. Doolan D. L. (2015). Large screen approaches to identify novel malaria vaccine candidates. Vaccine 33, 7496–7505. 10.1016/j.vaccine.2015.09.059, PMID: 26428458 PMC4729565

[ref22] Demarta-Gatsi C. Smith L. Thiberge S. Peronet R. Commere P. H. Matondo M.. (2016). Protection against malaria in mice is induced by blood stage-arresting histamine-releasing factor (HRF)-deficient parasites. J. Exp. Med. 213, 1419–1428. 10.1084/jem.20151976, PMID: 27432939 PMC4986535

[ref23] Doolan D. L. (2011). Plasmodium immunomics. Int. J. Parasitol. 41, 3–20. 10.1016/j.ijpara.2010.08.002, PMID: 20816843 PMC3005034

[ref24] Doolan D. L. Southwood S. Freilich D. A. Sidney J. Graber N. L. Shatney L.. (2003). Identification of *Plasmodium falciparum* antigens by antigenic analysis of genomic and proteomic data. Proc. Natl. Acad. Sci. U. S. A. 100, 9952–9957. 10.1073/pnas.1633254100, PMID: 12886016 PMC187898

[ref25] Douglas A. D. Baldeviano G. C. Lucas C. M. Lugo-Roman L. A. Crosnier C. Bartholdson S. J.. (2015). A PfRH5-based vaccine is efficacious against heterologous strain blood-stage *Plasmodium falciparum* infection in aotus monkeys. Cell Host Microbe 17, 130–139. 10.1016/j.chom.2014.11.017, PMID: 25590760 PMC4297294

[ref26] Douglas A. D. Williams A. R. Illingworth J. J. Kamuyu G. Biswas S. Goodman A. L.. (2011). The blood-stage malaria antigen PfRH5 is susceptible to vaccine-inducible cross-strain neutralizing antibody. Nat. Commun. 2:601. 10.1038/ncomms1615, PMID: 22186897 PMC3504505

[ref27] Elliott S. R. Kuns R. D. Good M. F. (2005). Heterologous immunity in the absence of variant-specific antibodies after exposure to subpatent infection with blood-stage malaria. Infect. Immun. 73, 2478–2485. 10.1128/IAI.73.4.2478-2485.2005, PMID: 15784594 PMC1087398

[ref28] EMA (2015). First Malaria Vaccine Receives Positive Scientific Opinion From EMA. Available at: https://www.ema.europa.eu/en/news/first-malaria-vaccine-receives-positive-scientificopinion-ema (Accessed January 10, 2021).

[ref29] Epstein J. E. Tewari K. Lyke K. E. Sim B. K. Billingsley P. F. Laurens M. B.. (2011). Live attenuated malaria vaccine designed to protect through hepatic CD8 T cell immunity. Science 334, 475–480. 10.1126/science.1211548, PMID: 21903775

[ref30] Fowkes F. J. Richards J. S. Simpson J. A. Beeson J. G. (2010). The relationship between anti-merozoite antibodies and incidence of *Plasmodium falciparum* malaria: a systematic review and meta-analysis. PLoS Med. 7:e1000218. 10.1371/journal.pmed.1000218, PMID: 20098724 PMC2808214

[ref31] Freund J. Thomson K. J. Sommer H. E. Walter A. W. Pisani T. M. (1948). Immunization of monkeys against malaria by means of killed parasites with adjuvants. Am. J. Trop. Med. Hyg. 28, 1–22. 10.4269/ajtmh.1948.s1-28.1, PMID: 18898694

[ref32] Fu Y. Lu X. Zhu F. Zhao Y. Ding Y. Ye L.. (2020). Improving the immunogenicity and protective efficacy of a whole-killed malaria blood-stage vaccine by chloroquine. Parasite Immunol. 42:e12682. 10.1111/pim.12682, PMID: 31644820

[ref33] Gardner M. J. Hall N. Fung E. White O. Berriman M. Hyman R. W.. (2002). Genome sequence of the human malaria parasite *Plasmodium falciparum*. Nature 419, 498–511. 10.1038/nature01097, PMID: 12368864 PMC3836256

[ref34] Goldman F. D. Gilman A. L. Hollenback C. Kato R. M. Premack B. A. Rawlings D. J. (2000). Hydroxychloroquine inhibits calcium signals in T cells: a new mechanism to explain its immunomodulatory properties. Blood 95, 3460–3466. 10.1182/blood.V95.11.3460, PMID: 10828029

[ref35] Good M. F. (2011). A whole parasite vaccine to control the blood stages of *Plasmodium*—the case for lateral thinking. Trends Parasitol. 27, 335–340. 10.1016/j.pt.2011.03.003, PMID: 21514227

[ref36] Good M. F. Reiman J. M. Rodriguez I. B. Ito K. Yanow S. K. El-Deeb I. M.. (2013). Cross-species malaria immunity induced by chemically attenuated parasites. J. Clin. Invest. 123, 3353–3362. 10.1172/JCI66634, PMID: 23863622 PMC4011145

[ref37] Hirunpetcharat C. Good M. F. (1998). Deletion of *Plasmodium berghei*-specific CD4+ T cells adoptively transferred into recipient mice after challenge with homologous parasite. Proc. Natl. Acad. Sci. U. S. A. 95, 1715–1720. 10.1073/pnas.95.4.1715, PMID: 9465082 PMC19161

[ref38] Julien J. P. Wardemann H. (2019). Antibodies against *Plasmodium falciparum* malaria at the molecular level. Nat. Rev. Immunol. 19, 761–775. 10.1038/s41577-019-0209-5, PMID: 31462718

[ref39] Junqueira C. Barbosa C. R. R. Costa P. A. C. Teixeira-Carvalho A. Castro G. Sen Santara S.. (2018). Cytotoxic CD8(+) T cells recognize and kill *Plasmodium vivax*-infected reticulocytes. Nat. Med. 24, 1330–1336. 10.1038/s41591-018-0117-4, PMID: 30038217 PMC6129205

[ref40] Keitany G. J. Kim K. S. Krishnamurty A. T. Hondowicz B. D. Hahn W. O. Dambrauskas N.. (2016). Blood stage malaria disrupts humoral immunity to the pre-erythrocytic stage circumsporozoite protein. Cell Rep. 17, 3193–3205. 10.1016/j.celrep.2016.11.060, PMID: 28009289 PMC5476299

[ref41] Kimura D. Miyakoda M. Kimura K. Honma K. Hara H. Yoshida H.. (2016). Interleukin-27-producing CD4(+) T cells regulate protective immunity during malaria parasite infection. Immunity 44, 672–682. 10.1016/j.immuni.2016.02.011, PMID: 26968425

[ref42] Kumar K. A. Sano G. Boscardin S. Nussenzweig R. S. Nussenzweig M. C. Zavala F.. (2006). The circumsporozoite protein is an immunodominant protective antigen in irradiated sporozoites. Nature 444, 937–940. 10.1038/nature05361, PMID: 17151604

[ref43] Kurup S. P. Obeng-Adjei N. Anthony S. M. Traore B. Doumbo O. K. Butler N. S.. (2017). Regulatory T cells impede acute and long-term immunity to blood-stage malaria through CTLA-4. Nat. Med. 23, 1220–1225. 10.1038/nm.4395, PMID: 28892065 PMC5649372

[ref44] Lambrecht B. N. Kool M. Willart M. A. Hammad H. (2009). Mechanism of action of clinically approved adjuvants. Curr. Opin. Immunol. 21, 23–29. 10.1016/j.coi.2009.01.004, PMID: 19246182

[ref45] Liu T. Xu G. Guo B. Fu Y. Qiu Y. Ding Y.. (2013). An essential role for C5aR signaling in the optimal induction of a malaria-specific CD4+ T cell response by a whole-killed blood-stage vaccine. J. Immunol. 191, 178–186. 10.4049/jimmunol.1201190, PMID: 23709683

[ref46] Low L. M. Ssemaganda A. Liu X. Q. Ho M. F. Ozberk V. Fink J.. (2019). Controlled infection immunization using delayed death drug treatment elicits protective immune responses to blood-stage malaria parasites. Infect. Immun. 87, e00587–e00618. 10.1128/IAI.00587-18, PMID: 30323025 PMC6300636

[ref47] Lu X. Liu T. Zhu F. Chen L. Xu W. (2017). A whole-killed, blood-stage lysate vaccine protects against the malaria liver stage. Parasite Immunol. 39:e12386. 10.1111/pim.12386, PMID: 27635936

[ref48] Lyke K. E. Ishizuka A. S. Berry A. A. Chakravarty S. DeZure A. Enama M. E.. (2017). Attenuated PfSPZ vaccine induces strain-transcending T cells and durable protection against heterologous controlled human malaria infection. Proc. Natl. Acad. Sci. U. S. A. 114, 2711–2716. 10.1073/pnas.1615324114, PMID: 28223498 PMC5347610

[ref49] Makobongo M. O. Riding G. Xu H. Hirunpetcharat C. Keough D. de Jersey J.. (2003). The purine salvage enzyme hypoxanthine guanine xanthine phosphoribosyl transferase is a major target antigen for cell-mediated immunity to malaria. Proc. Natl. Acad. Sci. U. S. A. 100, 2628–2633. 10.1073/pnas.0337629100, PMID: 12594331 PMC151391

[ref50] Moorthy V. S. Ballou W. R. (2009). Immunological mechanisms underlying protection mediated by RTS,S: a review of the available data. Malar. J. 8:312. 10.1186/1475-2875-8-312, PMID: 20042088 PMC2806383

[ref51] Mordmuller B. Surat G. Lagler H. Chakravarty S. Ishizuka A. S. Lalremruata A.. (2017). Sterile protection against human malaria by chemoattenuated PfSPZ vaccine. Nature 542, 445–449. 10.1038/nature21060, PMID: 28199305 PMC10906480

[ref52] Mueller A. K. Labaied M. Kappe S. H. Matuschewski K. (2005). Genetically modified *Plasmodium* parasites as a protective experimental malaria vaccine. Nature 433, 164–167. 10.1038/nature03188, PMID: 15580261

[ref53] Nahrendorf W. Scholzen A. Sauerwein R. W. Langhorne J. (2015a). Cross-stage immunity for malaria vaccine development. Vaccine 33, 7513–7517. 10.1016/j.vaccine.2015.09.098, PMID: 26469724 PMC4687527

[ref54] Nahrendorf W. Spence P. J. Tumwine I. Levy P. Jarra W. Sauerwein R. W.. (2015b). Blood-stage immunity to *Plasmodium chabaudi* malaria following chemoprophylaxis and sporozoite immunization. eLife 4:e05165. 10.7554/eLife.05165, PMID: 25714922 PMC4371380

[ref55] Niang M. Bei A. K. Madnani K. G. Pelly S. Dankwa S. Kanjee U.. (2014). STEVOR is a *Plasmodium falciparum* erythrocyte binding protein that mediates merozoite invasion and rosetting. Cell Host Microbe 16, 81–93. 10.1016/j.chom.2014.06.004, PMID: 25011110 PMC4382205

[ref56] Nussenzweig R. S. Vanderberg J. Most H. Orton C. (1967). Protective immunity produced by the injection of x-irradiated sporozoites of *Plasmodium berghei*. Nature 216, 160–162. 10.1038/216160a0, PMID: 6057225

[ref57] Obeng-Adjei N. Portugal S. Holla P. Li S. Sohn H. Ambegaonkar A.. (2017). Malaria-induced interferon-gamma drives the expansion of Tbethi atypical memory B cells. PLoS Pathog. 13:e1006576. 10.1371/journal.ppat.1006576, PMID: 28953967 PMC5633206

[ref58] Obeng-Adjei N. Portugal S. Tran T. M. Yazew T. B. Skinner J. Li S.. (2015). Circulating Th1-cell-type Tfh cells that exhibit impaired B cell help are preferentially activated during acute malaria in children. Cell Rep. 13, 425–439. 10.1016/j.celrep.2015.09.004, PMID: 26440897 PMC4607674

[ref59] Ocana-Morgner C. Mota M. M. Rodriguez A. (2003). Malaria blood stage suppression of liver stage immunity by dendritic cells. J. Exp. Med. 197, 143–151. 10.1084/jem.20021072, PMID: 12538654 PMC2193811

[ref60] Ogutu B. R. Apollo O. J. McKinney D. Okoth W. Siangla J. Dubovsky F.. (2009). Blood stage malaria vaccine eliciting high antigen-specific antibody concentrations confers no protection to young children in Western Kenya. PLoS One 4:e4708. 10.1371/journal.pone.0004708, PMID: 19262754 PMC2650803

[ref61] O’Meara W. P. Mangeni J. N. Steketee R. Greenwood B. (2010). Changes in the burden of malaria in sub-Saharan Africa. Lancet Infect. Dis. 10, 545–555. 10.1016/S1473-3099(10)70096-7, PMID: 20637696

[ref62] Phyo A. P. Nkhoma S. Stepniewska K. Ashley E. A. Nair S. McGready R.. (2012). Emergence of artemisinin-resistant malaria on the western border of Thailand: a longitudinal study. Lancet 379, 1960–1966. 10.1016/S0140-6736(12)60484-X, PMID: 22484134 PMC3525980

[ref63] Pinzon-Charry A. McPhun V. Kienzle V. Hirunpetcharat C. Engwerda C. McCarthy J.. (2010). Low doses of killed parasite in CpG elicit vigorous CD4+ T cell responses against blood-stage malaria in mice. J. Clin. Invest. 120, 2967–2978. 10.1172/JCI39222, PMID: 20628205 PMC2912178

[ref64] Pinzon-Charry A. Woodberry T. Kienzle V. McPhun V. Minigo G. Lampah D. A.. (2013). Apoptosis and dysfunction of blood dendritic cells in patients with falciparum and vivax malaria. J. Exp. Med. 210, 1635–1646. 10.1084/jem.20121972, PMID: 23835848 PMC3727318

[ref65] Pombo D. J. Lawrence G. Hirunpetcharat C. Rzepczyk C. Bryden M. Cloonan N.. (2002). Immunity to malaria after administration of ultra-low doses of red cells infected with *Plasmodium falciparum*. Lancet 360, 610–617. 10.1016/S0140-6736(02)09784-2, PMID: 12241933

[ref66] Prinz H. Sattler J. M. Roth A. Ripp J. Adams J. H. Frischknecht F. (2018). Immunization efficacy of cryopreserved genetically attenuated *Plasmodium berghei* sporozoites. Parasitol. Res. 117, 2487–2497. 10.1007/s00436-018-5937-0, PMID: 29797085

[ref67] Rai D. Pham N. L. Harty J. T. Badovinac V. P. (2009). Tracking the total CD8 T cell response to infection reveals substantial discordance in magnitude and kinetics between inbred and outbred hosts. J. Immunol. 183, 7672–7681. 10.4049/jimmunol.0902874, PMID: 19933864 PMC2808048

[ref68] Raja A. I. Cai Y. Reiman J. M. Groves P. Chakravarty S. McPhun V.. (2016). Chemically attenuated blood-stage *Plasmodium yoelii* parasites induce long-lived and strain-transcending protection. Infect. Immun. 84, 2274–2288. 10.1128/IAI.00157-16, PMID: 27245410 PMC4962623

[ref69] Ranson H. N’Guessan R. Lines J. Moiroux N. Nkuni Z. Corbel V. (2011). Pyrethroid resistance in African anopheline mosquitoes: what are the implications for malaria control? Trends Parasitol. 27, 91–98. 10.1016/j.pt.2010.08.004, PMID: 20843745

[ref70] Rathore D. Sacci J. B. de la Vega P. McCutchan T. F. (2002). Binding and invasion of liver cells by *Plasmodium falciparum* sporozoites. Essential involvement of the amino terminus of circumsporozoite protein. J. Biol. Chem. 277, 7092–7098. 10.1074/jbc.M106862200, PMID: 11751898

[ref71] Roestenberg M. McCall M. Hopman J. Wiersma J. Luty A. J. van Gemert G. J.. (2009). Protection against a malaria challenge by sporozoite inoculation. N. Engl. J. Med. 361, 468–477. 10.1056/NEJMoa0805832, PMID: 19641203

[ref72] Roestenberg M. Walk J. van der Boor S. C. Langenberg M. C. C. Hoogerwerf M. A. Janse J. J.. (2020). A double-blind, placebo-controlled phase 1/2a trial of the genetically attenuated malaria vaccine PfSPZ-GA1. Sci. Transl. Med. 12:eaaz5629. 10.1126/scitranslmed.aaz5629, PMID: 32434847

[ref73] Rts S. C. T. P. (2015). Efficacy and safety of RTS,S/AS01 malaria vaccine with or without a booster dose in infants and children in Africa: final results of a phase 3, individually randomised, controlled trial. Lancet 386, 31–45. 10.1016/S0140-6736(15)60721-8, PMID: 25913272 PMC5626001

[ref74] Ryg-Cornejo V. Ioannidis L. J. Ly A. Chiu C. Y. Tellier J. Hill D. L.. (2016). Severe malaria infections impair germinal center responses by inhibiting T follicular helper cell differentiation. Cell Rep. 14, 68–81. 10.1016/j.celrep.2015.12.006, PMID: 26725120

[ref75] Sack B. K. Keitany G. J. Vaughan A. M. Miller J. L. Wang R. Kappe S. H. (2015). Mechanisms of stage-transcending protection following immunization of mice with late liver stage-arresting genetically attenuated malaria parasites. PLoS Pathog. 11:e1004855. 10.1371/journal.ppat.1004855, PMID: 25974076 PMC4431720

[ref76] Sagara I. Dicko A. Ellis R. D. Fay M. P. Diawara S. I. Assadou M. H.. (2009). A randomized controlled phase 2 trial of the blood stage AMA1-C1/Alhydrogel malaria vaccine in children in Mali. Vaccine 27, 3090–3098. 10.1016/j.vaccine.2009.03.014, PMID: 19428923 PMC2713037

[ref77] Sagara I. Healy S. A. Assadou M. H. Gabriel E. E. Kone M. Sissoko K.. (2018). Safety and immunogenicity of Pfs25H-EPA/alhydrogel, a transmission-blocking vaccine against *Plasmodium falciparum*: a randomised, double-blind, comparator-controlled, dose-escalation study in healthy Malian adults. Lancet Infect. Dis. 18, 969–982. 10.1016/S1473-3099(18)30344-X, PMID: 30061051 PMC6287938

[ref78] Schussek S. Trieu A. Apte S. H. Sidney J. Sette A. Doolan D. L. (2017). Novel *Plasmodium* antigens identified via genome-based antibody screen induce protection associated with polyfunctional T cell responses. Sci. Rep. 7:15053. 10.1038/s41598-017-15354-0, PMID: 29118376 PMC5678182

[ref79] Seder R. A. Chang L. J. Enama M. E. Zephir K. L. Sarwar U. N. Gordon I. J.. (2013). Protection against malaria by intravenous immunization with a nonreplicating sporozoite vaccine. Science 341, 1359–1365. 10.1126/science.1241800, PMID: 23929949

[ref80] Sirima S. B. Cousens S. Druilhe P. (2011). Protection against malaria by MSP3 candidate vaccine. N. Engl. J. Med. 365, 1062–1064. 10.1056/NEJMc1100670, PMID: 21916656

[ref81] Sissoko M. S. Healy S. A. Katile A. Omaswa F. Zaidi I. Gabriel E. E.. (2017). Safety and efficacy of PfSPZ vaccine against *Plasmodium falciparum* via direct venous inoculation in healthy malaria-exposed adults in Mali: a randomised, double-blind phase 1 trial. Lancet Infect. Dis. 17, 498–509. 10.1016/S1473-3099(17)30104-4, PMID: 28216244 PMC6803168

[ref82] Smith T. Ross A. Maire N. Chitnis N. Studer A. Hardy D.. (2012). Ensemble modeling of the likely public health impact of a pre-erythrocytic malaria vaccine. PLoS Med. 9:e1001157. 10.1371/journal.pmed.1001157, PMID: 22272189 PMC3260300

[ref83] Spring M. Murphy J. Nielsen R. Dowler M. Bennett J. W. Zarling S.. (2013). First-in-human evaluation of genetically attenuated *Plasmodium falciparum* sporozoites administered by bite of *Anopheles* mosquitoes to adult volunteers. Vaccine 31, 4975–4983. 10.1016/j.vaccine.2013.08.007, PMID: 24029408

[ref84] Stanisic D. I. Fink J. Mayer J. Coghill S. Gore L. Liu X. Q.. (2018). Vaccination with chemically attenuated *Plasmodium falciparum* asexual blood-stage parasites induces parasite-specific cellular immune responses in malaria-naive volunteers: a pilot study. BMC Med. 16:184. 10.1186/s12916-018-1173-9, PMID: 30293531 PMC6174572

[ref85] Sutherland C. (2007). A challenge for the development of malaria vaccines: polymorphic target antigens. PLoS Med. 4:e116. 10.1371/journal.pmed.0040116, PMID: 17388671 PMC1820606

[ref86] Tarun A. S. Peng X. Dumpit R. F. Ogata Y. Silva-Rivera H. Camargo N.. (2008). A combined transcriptome and proteome survey of malaria parasite liver stages. Proc. Natl. Acad. Sci. U. S. A. 105, 305–310. 10.1073/pnas.0710780104, PMID: 18172196 PMC2224207

[ref87] Thera M. A. Doumbo O. K. Coulibaly D. Laurens M. B. Ouattara A. Kone A. K.. (2011). A field trial to assess a blood-stage malaria vaccine. N. Engl. J. Med. 365, 1004–1013. 10.1056/NEJMoa1008115, PMID: 21916638 PMC3242358

[ref88] Ting L. M. Gissot M. Coppi A. Sinnis P. Kim K. (2008). Attenuated *Plasmodium yoelii* lacking purine nucleoside phosphorylase confer protective immunity. Nat. Med. 14, 954–958. 10.1038/nm.1867, PMID: 18758447 PMC3937818

[ref89] Urban B. C. Ferguson D. J. Pain A. Willcox N. Plebanski M. Austyn J. M.. (1999). *Plasmodium falciparum*-infected erythrocytes modulate the maturation of dendritic cells. Nature 400, 73–77. 10.1038/21900, PMID: 10403251

[ref90] Uwimana A. Legrand E. Stokes B. H. Ndikumana J. M. Warsame M. Umulisa N.. (2020). Emergence and clonal expansion of in vitro artemisinin-resistant *Plasmodium falciparum* kelch13 R561H mutant parasites in Rwanda. Nat. Med. 26, 1602–1608. 10.1038/s41591-020-1005-2, PMID: 32747827 PMC7541349

[ref91] Vaughan A. M. Wang R. Kappe S. H. (2010). Genetically engineered, attenuated whole-cell vaccine approaches for malaria. Hum. Vaccin. 6, 107–113. 10.4161/hv.6.1.9654, PMID: 19838068 PMC3641786

[ref92] Vijay R. Guthmiller J. J. Sturtz A. J. Surette F. A. Rogers K. J. Sompallae R. R.. (2020). Infection-induced plasmablasts are a nutrient sink that impairs humoral immunity to malaria. Nat. Immunol. 21, 790–801. 10.1038/s41590-020-0678-5, PMID: 32424361 PMC7316608

[ref93] Volz J. C. Yap A. Sisquella X. Thompson J. K. Lim N. T. Whitehead L. W.. (2016). Essential role of the PfRh5/PfRipr/CyRPA complex during *Plasmodium falciparum* invasion of erythrocytes. Cell Host Microbe 20, 60–71. 10.1016/j.chom.2016.06.004, PMID: 27374406

[ref94] Wahlgren M. Goel S. Akhouri R. R. (2017). Variant surface antigens of *Plasmodium falciparum* and their roles in severe malaria. Nat. Rev. Microbiol. 15, 479–491. 10.1038/nrmicro.2017.47, PMID: 28603279

[ref95] Weiss G. E. Crompton P. D. Li S. Walsh L. A. Moir S. Traore B.. (2009). Atypical memory B cells are greatly expanded in individuals living in a malaria-endemic area. J. Immunol. 183, 2176–2182. 10.4049/jimmunol.0901297, PMID: 19592645 PMC2713793

[ref96] Weiss G. E. Gilson P. R. Taechalertpaisarn T. Tham W. H. de Jong N. W. Harvey K. L.. (2015). Revealing the sequence and resulting cellular morphology of receptor-ligand interactions during *Plasmodium falciparum* invasion of erythrocytes. PLoS Pathog. 11:e1004670. 10.1371/journal.ppat.1004670, PMID: 25723550 PMC4344246

[ref97] WHO (2019). MVIP Countries: Ghana, Kenya, and Malawi. Available at: https://www.who.int/immunization/diseases/malaria/malaria_vaccine_implementation_programme/pilot_countries_ghana_kenya_malawi/en/ (Accessed January 10, 2021).

[ref98] Wipasa J. Xu H. Stowers A. Good M. F. (2001). Apoptotic deletion of Th cells specific for the 19-kDa carboxyl-terminal fragment of merozoite surface protein 1 during malaria infection. J. Immunol. 167, 3903–3909. 10.4049/jimmunol.167.7.3903, PMID: 11564808

[ref99] Woodberry T. Minigo G. Piera K. A. Amante F. H. Pinzon-Charry A. Good M. F.. (2012). Low-level *Plasmodium falciparum* blood-stage infection causes dendritic cell apoptosis and dysfunction in healthy volunteers. J. Infect. Dis. 206, 333–340. 10.1093/infdis/jis366, PMID: 22615323

[ref100] Woodberry T. Pinzon-Charry A. Piera K. A. Panpisutchai Y. Engwerda C. R. Doolan D. L.. (2009). Human T cell recognition of the blood stage antigen *Plasmodium* hypoxanthine guanine xanthine phosphoribosyl transferase (HGXPRT) in acute malaria. Malar. J. 8:122. 10.1186/1475-2875-8-122, PMID: 19500406 PMC2700129

[ref101] Wright K. E. Hjerrild K. A. Bartlett J. Douglas A. D. Jin J. Brown R. E.. (2014). Structure of malaria invasion protein RH5 with erythrocyte basigin and blocking antibodies. Nature 515, 427–430. 10.1038/nature13715, PMID: 25132548 PMC4240730

[ref102] Wu Y. Ellis R. D. Shaffer D. Fontes E. Malkin E. M. Mahanty S.. (2008). Phase 1 trial of malaria transmission blocking vaccine candidates Pfs25 and Pvs25 formulated with montanide ISA 51. PLoS One 3:e2636. 10.1371/journal.pone.0002636, PMID: 18612426 PMC2440546

[ref103] Wykes M. N. Zhou Y. H. Liu X. Q. Good M. F. (2005). *Plasmodium yoelii* can ablate vaccine-induced long-term protection in mice. J. Immunol. 175, 2510–2516. 10.4049/jimmunol.175.4.2510, PMID: 16081823

[ref104] Xu H. Wipasa J. Yan H. Zeng M. Makobongo M. O. Finkelman F. D.. (2002). The mechanism and significance of deletion of parasite-specific CD4(+) T cells in malaria infection. J. Exp. Med. 195, 881–892. 10.1084/jem.20011174, PMID: 11927632 PMC2193727

[ref105] Zhu F. Liu T. Zhao C. Lu X. Zhang J. Xu W. (2017). Whole-killed blood-stage vaccine-induced immunity suppresses the development of malaria parasites in mosquitoes. J. Immunol. 198, 300–307. 10.4049/jimmunol.1600979, PMID: 27903741

